# Knowledge, attitude and preventive practice towards COVID-19 and associated factors among outpatient service visitors at Debre Markos compressive specialized hospital, north-west Ethiopia, 2020

**DOI:** 10.1371/journal.pone.0251708

**Published:** 2021-07-15

**Authors:** Bekele Taye Feleke, Mengistu Zelalem Wale, Mesenbet Terefe Yirsaw

**Affiliations:** 1 Department of Medicine, School of Medicine, Debre Markos University, Debre Markos, Ethiopia; 2 Department of Biomedical Sciences, School of Medicine, Debre Markos University, Debre Markos, Ethiopia; All India Institute of Medical Sciences Jodhpur, INDIA

## Abstract

**Background:**

COVID-19 is an emerging infectious disease which is a major public health problem worldwide. Given the serious threats imposed by COVID-19 and the absence of vaccines until August 2020, preventive measures play an essential role in reducing infection rates and controlling its spread. This shows the necessity of public adherence for preventive and control measures, which is affected by their knowledge, attitudes, and practices.

**Objective:**

This study aimed to determine knowledge, attitude, and practice (KAP) towards COVID-19 and associated factors among outpatient service visitors, Debre Markos compressive specialized hospital, north-west,Ethiopia.

**Method:**

Institutional-based cross-sectional study design with a systematic random sampling technique was conducted from July to August 2020. Among a total of 404 participants, 398 were recruited. Data were collected using a structured questionnaire. The data was edited, coded, and entered into Epi data version 4.6 and exported to SPSS-25 for analysis. Bivariable and multivariable logistic regression models were employed to identify factors associated with KAP. A p-value of <0.05 was considered statistically significant.

**Result:**

The prevalence of poor knowledge, attitude and practice among the outpatient service visitors were 27.1%, 30.7% and 44%, respectively. The mean age of the participants was 33.4 ±10.9 years. Variables like; educational status, ‘‘can’t read and write” [AOR = 3.76, 95% CI (1.36–10.42), P = 0.01], read and write [AOR = 5.90, 95% CI (2.39–14.98), P = 0.01], rural residence [AOR = 3.04, 95% CI (1.43–6.46), P = 0.01] and having no television [AOR = 0.8, 95% CI (0.79–0.89), P = 0.03] were significantly associated with poor knowledge. While, educational status of “can’t read and write”, [AOR = 6.71, 95% CI (2.78–16.16), P = 0.01] and rural residence [AOR = 2.03, 95% CI (1.14–3.61), P = 0.02] were significantly associated with poor attitude. Additionally, poor knowledge, [AOR = 22.73, 95% CI (10.5–49.21), P = 0.01], rural residence [AOR = 2.08, 95% CI (1.08–4.88), P = 0.04] and having no television [AOR = 2.24, 95% CI (1.05–4.79), P = 0.01] were significantly associated with poor practice.

**Conclusion:**

In this study, knowledge, attitude, and practice among outpatient service visitors was poor which needs targeted health education and interventions from the health professional to enhance their knowledge, attitude, and practice towards COVID-19. In parallel with this, special attention should be given for the rural community and for those with an educational status of can’t read and write.

## Background

Coronavirus disease 2019 (COVID-19) is a type of infectious disease caused by a novel virus to be included in the virus group, coronaviridae. It was first identified in late December 2019 in China, and has since spread globally, resulting in the ongoing 2019–20 coronavirus pandemic [1]. Research findings showed that, coronavirus spreads from human-to-human, mainly through respiratory droplets, and contact with contaminated materials with hands, and touching of faces-eye-nose-mouth are major ways to be exposed to the virus [2–4]. The clinical symptoms of COVID-19 include; fever, cough, fatigue, malaise, and shortness of breath [5]. Until March, 15, 2020 there was no confirmed treatment or vaccination against the virus, SARS-CoV-2. Due to this public awareness about the virus plays a critical role to minimize its spread, especially in low-income countries including Ethiopia where health facilities are poorely developed [6].

As of December 2019 until August 2020, the pandemics registered 21, 368, 534 cases, and 763,572 deaths in the world and 925, 297 cases, and 17,904 deaths in Africa giving a crude fatality ratio of 3.4% in the worldwide and 1.94% in Africa [7]. Ethiopia has become among the COVID-19 affected countries as of March 15, 2020, the date on which one imported case was first detected. According to the Ministry of Health (MOH) information on August 14, 2020, there were 27,242 total notified cases and 492 deaths in giving a crude fatality rate of 1.8% in Ethiopia [8].

Evidence shows that public knowledge is important in tackling the pandemics [9,10]. By assessing public awareness and knowledge about the virus, deeper insights into the existing public perception and practices can be gained, thereby helping to identify attributes that influence the public in adopting healthy practices and responsive behavior [11].

Studies analyzing attitudes and knowledge about COVID-19 concluded that, attitude towards government measures related to minimizing the spread of the epidemic were highly associated with the level of knowledge about COVID-19 [12]. Studies reported that higher levels of information and education were associated with more positive attitude and preventive practice towards COVID-19 [12]. Even though there are strong initiatives and recognition of the public health importance against COVID-19 by the Ethiopian government (screening, quarantine, and treatment centers), there is a strong need to reinforce community KAP to control the spread of the virus.

Since there were limited studies in the country and no scientific evidence of such a study in this study area, it is crucial to determine the level of knowledge, attitude, and preventive practice towards COVID-19 and its associated factors among outpatient service visitors in Debre Markos compressive specialized Hospital, Amhara regional state, north-west Ethiopia, 2020. Determining the magnitude will serve as further public health interventions, awareness, and policy improvements on the COVID-19. This study will be used as an awakening reference for further and deeper investigation regarding the importance of community preventive measures towards COVID-19.

## Methods

### Study setting

The study was conducted at Debre Markos comprehensive specialized hospital from July to August 2020. The hospital is found in Debre Markos town, East Gojjam Zone, Amhara regional state, which is 299 km North West of Addis Ababa, the capital city of Ethiopia, and 268 Km southwest of Bahir Dar, the capital city of Amhara regional state. The Hospital was built in 1957 to serve about 25,000 people but now it is serving more than 5 million people.

### Study design and population

An institutional-based cross-sectional study design with a systematic random sampling technique was conducted. All patients who attended the outpatient department (OPD) of Debre Markos comprehensive specialized hospital were the source population and all patients fulfilling the inclusion criteria during the study period were the study population. All participants who were mentally or physically capable of giving consent, those who were willing to participate, and those whose age was 18 years and above were included while those who were critically and acutely ill during the data collection period were excluded. Since there were no similar studies related to KAP towards COVID-19 in the study area, the sample size was calculated using single population proportion formula based on the assumption that the probability of having poor knowledge, attitude and preventive practice towards COVID-19 was 50.0%, at 95% CI, 5% margin of error; and determined to be 384. By adding non-response rate of 5%, the total sample size was 404.

### Data collection tool and procedure

Before starting the study, participants were informed about their full right to refuse. During data collection, the reasonable physical distance was kept between the participants and the data collector. The data was collected in a private condition and keep confidential. During data collection, information about COVID-19 was provided for patients after completed data collection. The total sample size was allocated to each outpatient department based on the probability proportional to patient flow number in each department. By dividing the daily patient flow to the number of patients to be surveyed per day, every fifth patient was approached to the interview. The average daily patients flow to the OPD was estimated to be 214 and the average number of patients to be interviewed every ten days of data collection was 40. The first patient was selected daily by drawing a number from 1 to 5 by using the lottery method. Investigators administered close-ended questionnaires were prepared by adopting from related studies to measure KAP towards COVID-19 [13,14]. The questionnaire consisted of four main areas: 1) Sociodemographic characteristics, 2) knowledge about COVID-19; 3) Attitudes toward COVID-19; and 4) practices relevant to COVID-19. To measure knowledge about COVID-19, 13 items were adapted from previous research [14]. These items include assessing the participants’ knowledge about clinical presentations (items 1–4), transmission routes (items 5–8), and prevention and control (items 9–13) of COVID-19. Participants were given “yes,” “no,” or “I don’t know” response options to these items.

A correct response to an item will be assigned 1 point, while an incorrect/I don’t know response/were assigned 0 points.

To measure attitudes towards COVID-19, surveyed participants were asked whether they agreed, disagreed, or were not sure. To measure practices, participants were asked yes/no questions, the correct answer was assigned 1 point and the incorrect answer was assigned 0 points. The reliability of the knowledge, attitude, and practice questionnaires was checked and the values of Cronbach’s alpha were 0.71, 0.78, and 0.76 respectively, indicating acceptable internal consistency. There were 4 clinical nurse data collectors participate to collect the data. The overall data collection process was coordinated and supervised by the principal supervisors. The filled questionnaires were checked by the supervisors for completeness and consistency of responses.

### Ethical consideration

Before data collection, a letter of Ethical clearance was obtained from Debre Markos University, School of Medicine, and official permission was collected from Debre Markos Comprehensive specialized hospital. Verbal informed consent was obtained from the participants. Since there was no clinical intervention that may harm the study participants, in order to avoid contact across the participants and the data collectors due to the COVID-19 pandemic, and some of the study participants were unable to write and read; we were forced to use verbal informed consent.

### Data analysis and statistics

After the data was collected, it was checked for inconsistency and eligibility issues. The collected data were entered into Epi data version 4.6 for cross-checking and exported to SPSS version 25.0 for analysis. Descriptive statistics were used to describe the data. During the analysis, frequencies of different variables were determined, followed by cross-tabulation to compare the frequencies. Binary logistic regression was used to assess the association between independent and dependent variables. All variables that were showed statistical significance in the bivariate analysis with a P-value of ≤0.2, were entered into a multi-variety logistic regression model to determine the independent factors associated with poor KAP among outpatient service visitors. A p-value of <0.05 was considered statistically significant.

### Operational definition

- The overall **knowledge** was categorized, using Bloom’s cut-off point, as good if the score was between 80 and 100%, moderate if the score was between 60 and 79%, and poor if the score was less than 60% [13].
- The overall **attitude** was categorized, using Bloom’s cut-off point, as good if the score was between 80 and 100%, moderate if the score was between 60 and 79%, and poor if the score was less than 60% [13].
- The overall **practice** score will be categorized using the same Bloom’s cut-off point, as good if the score will be between 80 and 100%, moderate if the score will be between 60 and 79%, and poor if the score will be less than 60% [13].

## Results

### Sociodemographic characteristics

Out of a total sample size of 404, 398 participants were participated in this study giving a response rate of 98.5%. From this, 223(56%) were males, and 175 (44%) were females. The mean age of the participants was 33.4 ±10.9 SD years. Most, 353 (88.7%) of the participants were the follower of Orthodox Christian religion. More than half, 274 (68.8%) of the participants were from an urban area while, 124(31.2%) of them were from the rural area. From the participants, 73 (18.3%) of the them can’t read and write while 92, (23.1%) had “college and above” educational level (**Table 1).**

**Table 1:** Socio-demographic characteristics of the outpatient service visitors in Debre Markos compressive specialized hospital, Amhara region, Ethiopia, 2020.

Variables	Category	Frequency	Percentage
Age	18–29 years	174	43.7
	30–39 years	113	28.4
	40–49 years	74	18.6
	50–59 years	29	7.3
	≥60 years	8	2.0
Sex	Male	223	56.0
	Female	175	44.0
Religion	Orthodox	353	88.7
	Muslim	41	10.3
	Protestant	4	1.0
Residence	Urban	274	68.8
	Rural	124	31.2
Marital status	Single	91	22.9
	Married	290	72.9
	Widowed	10	2.5
	Divorce	7	1.8
Educational status	Unable to read and write	73	18.3
	Read and write	82	20.6
	Elementary (1–8)	94	23.6
	Secondary (9–12)	57	14.3
	College and above	92	23.1
Occupation	Daily laborer	21	5.3
	Merchant	92	23.1
	Private employee/business	76	19.1
	Government employee	91	22.8
	Farmer	89	22.4
	Others*	29	7.3
Monthly income (ETB)	≤1000 ETB	103	25.9
	1001–2500 ETB	109	27.4
	≥2600 ETB	186	46.7

Others *(students and housewife).

### Sources of information for the participants

**Fig 1:**
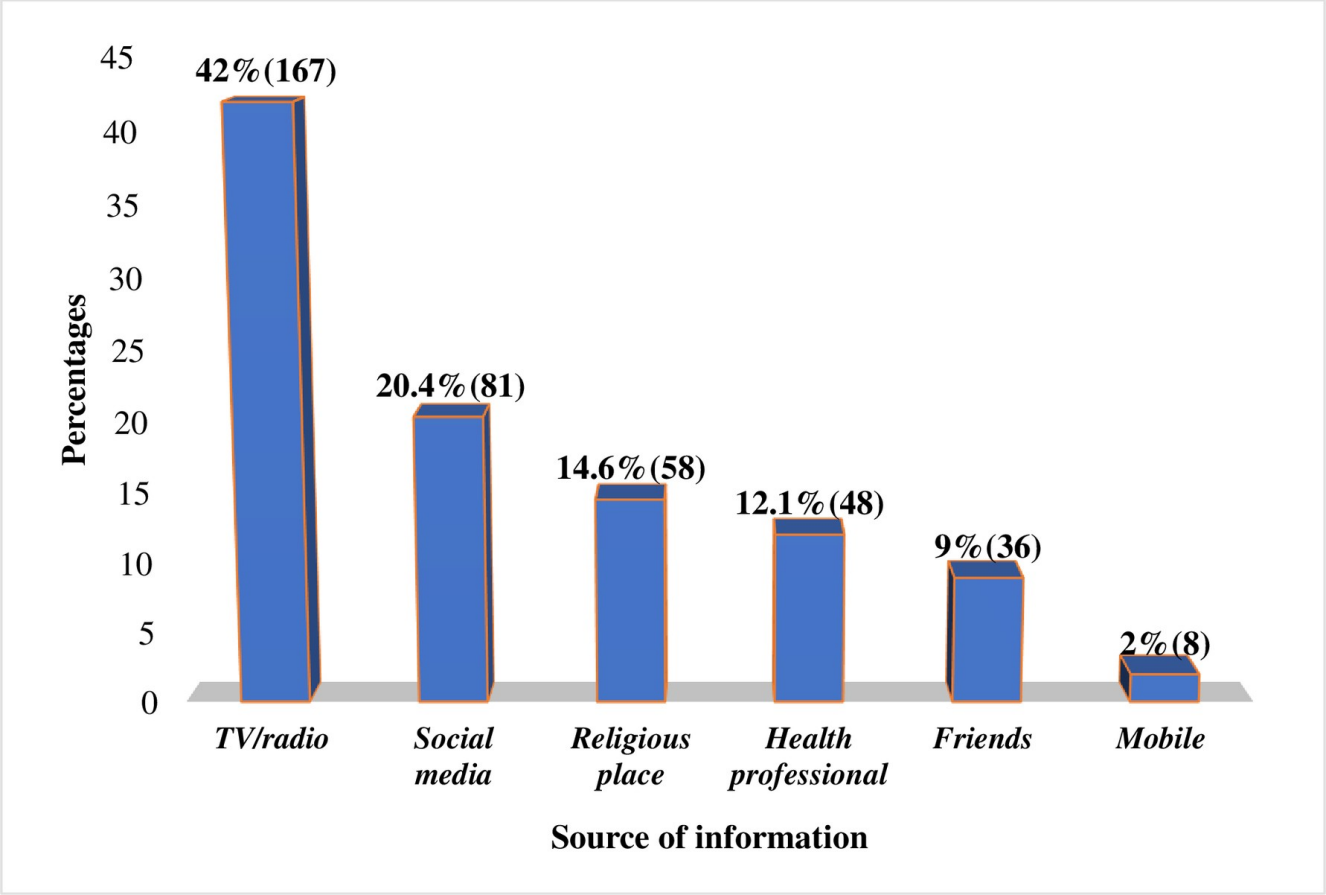
Source of information about COVID-19 among the outpatient service visitors at Debre Markos compressive specialized hospital, Amhara regional state, Ethiopia, 2020.

All participants had information on COVID-19. TV/radio, 167 (42%) and social media, 81 (20.4%) were the most commonly stated sources of information. Among the participants, 226 (56.8%) and 100 (25.1%) of them have television and radio respectively **(Fig 1).**

### Knowledge of the participants about COVID-19

**Fig 2:**
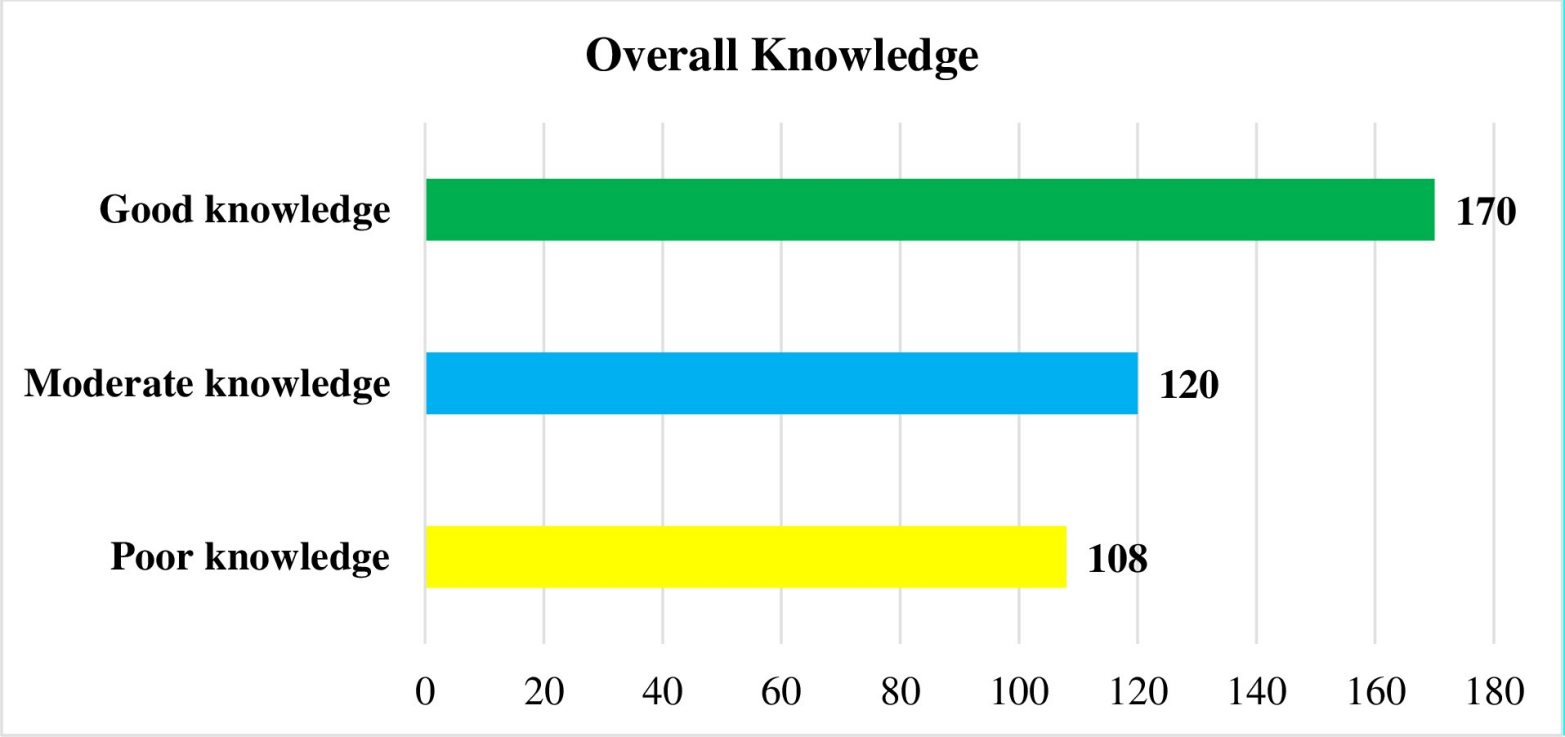
Level of overall knowledge among outpatient service visitors on COVID-19 at Debre Markos compressive specialized hospital, Amhara regional state, Ethiopia, 2020.

The overall knowledge of the participants towards COVID-19 indicated that 108 (27.1%) had poor knowledge while 170 (42.7%) of them had good knowledge **(Fig 2).**

The average knowledge score of the participants was 9.2 (SD = 2.8, ranging from 3–13). About, 290, (72.9%) of the participants scored above 8, representing an acceptable level of knowledge on COVID-19. Out of the participants, 390 (90.5%) of them knew the main clinical symptoms of COVID-19 as fever, fatigue, dry cough, and myalgia. About, 212 (53.3%) of the participants correctly answered symptoms such as; stuffy nose, runny nose, and sneezing, which distinguishes COVID-19 from common cold/flu **(Table 2)**.

**Table 2:** Knowledge towards COVID-19 among outpatient service visitors at Debre Markos comprehensive specialized hospital, Amhara regional state, Ethiopia, 2020.

S. No	Knowledge related questions	Frequency (%)		
		Yes	No	Don’t Know
		n (%)	n (%)	n (%)
1.	The main clinical symptoms of COVID-19 are fever, cough, shortness of breath, and fatigue.	390(90.5)	30(7.5)	8(2)
2.	Unlike the common cold, stuffy nose, runny nose, and sneezing are less common in persons infected with the COVID-19 virus.	212(53.3)	176(44.2)	10(2.5)
3.	Currently, there is no effective cure for COVID-19, but early symptomatic and supportive treatment can help most patients recover from the infection.	323(81.2)	51(12.8)	24(6)
4.	Not all persons with COVID-19 will develop into severe cases. Only those who are elderly and have chronic illnesses are more likely to be severe cases.	303(76.1)	63(15.8)	24(6)
5.	Eating or touching wild animals would result in the infection by the COVID-19 virus.	218(54.8)	42(10.6)	138(34.7)
6.	Persons with COVID-19 can’t infect the virus to others if they do not have a fever.	176(44.2)	172(43.2)	50(12.6)
7.	COVID-19 virus spreads via respiratory droplets of infected individuals.	311(78.1)	51(12.8)	36(9)
8.	One way of prevention of COVID-19 is not touching the eye, nose by unwashed hands.	342(85.9)	45(11.3)	11(2.8)
9.	Ordinary residents can wear face masks to prevent the infection.	343(86.2)	42(10.6)	13(3.3)
10.	Children and young adults do not need to take measures to prevent the infection by the COVID-19 virus.	144(36.2)	216(54.3)	38(9.5)
11.	To prevent infection by COVID-19, an individual should avoid going to crowded places.	305(76.6)	79(19.8)	14(3.5)
12.	Isolation and treatment of people who are infected with the COVID-19 virus are effective ways to reduce the spread of the virus.	317(79.6)	67(16.8)	14(3.5)
13.	People who have contact with someone infected with the COVID-19 virus should be immediately isolated in a proper place.	314(78.9)	70(17.6)	14(3.5)

### Factors associated with poor knowledge

Those participants whose educational status was below elemenatary educational level were 3 to 5 times more likely to have poor knowledge than those with educational status of “college and above”. The odds of having poor knowledge in rural residents were 3 times [AOR = 3.04, 95% CI (1.43–6.46)] **(Table 3).**

**Table 3:** Factors associated with poor knowledge among outpatient service visitors at Debre Markos comprehensive specialized hospital, Amhara regional state, Ethiopia, 2020.

Variables	Poor knowledge		COR (95%CI)	AOR (95%CI)	p-value
	Yes	No			
Educational status					
Unable to read and write	36	37	7.17(3.28–15.6)	**3.76(1.36–10.42)**	0.01*
Read and write	42	40	7.73(3.60–16.60)	**5.90(2.39–14.98)**	0.01*
Elementary (1–8)	18	76	1.74(0.77–3.93)	1.61(0.68–3.85)	0.28
Secondary (9–12)	5	52	0.70 (0.12–1.05)	0.14(0.02–1.15)	0.07
College and above	11	81	1	1	
Residence					
Urban	38	236	1	1	
Rural	70	54	8.05(4.92–13.19)	**3.04(1.43–6.46)**	0.01*
Monthly income					
≤1000 ETB	40	63	1.99(1.18–3.34)	1.36 (0.17–1.76)	0.07
1001–2500 ETB	23	86	0.84(0.47–1.48)	0.23(0.11–1.22)	0.06
≥2600 ETB	45	141	1	1	
Having television					
Yes	28	198	1	1	
No	80	92	6.15(3.74–10.1)	**2.24(1.05–4.79)**	0.03*

Note: *Statistically significant.

### The attitude of outpatient service visitors towards COVID-19

**Fig 3:**
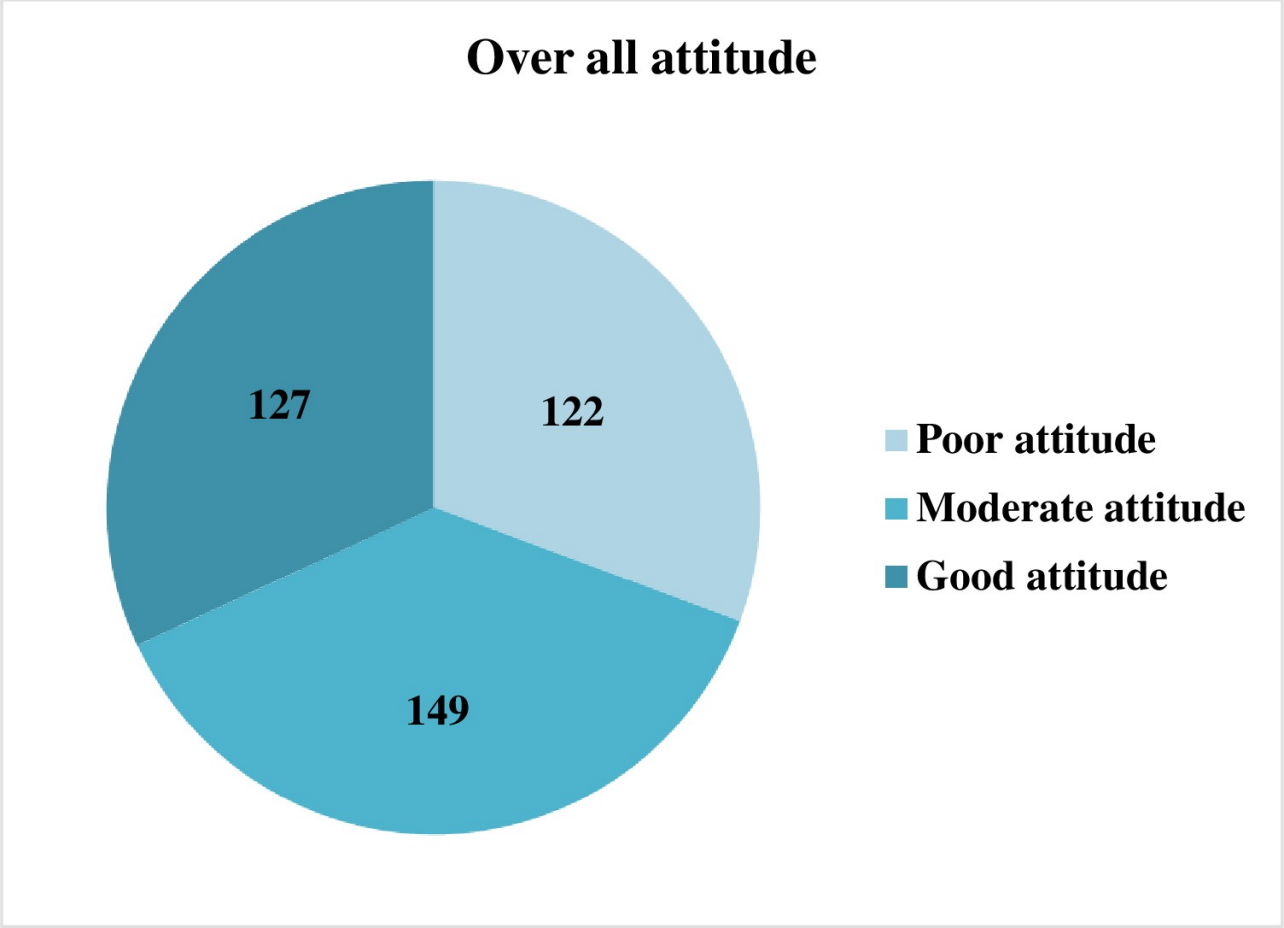
Level of overall attitude towards COVID-19 among outpatient service visitors at Debre Markos compressive specialized hospital, Amhara regional state, Ethiopia, 2020.

The overall attitude of the participants towards COVID-19 indicated that 122 (30.7%), 149 (37.4%), and 127 (31.9%) of them had a poor, moderate and good attitude, respectively **(Fig 3).**

Half, 199 (50%) of participants agreed that COVID-19 would successfully be controlled. Only 135 (33.9%) of the participants had agreed that strict measures would be able to win the battle against COVID-19, while, 60 (15.1%) did not agree **(Table 4).**

**Table 4:** Attitude towards COVID-19 among outpatient service visitors at Debre Markos comprehensive specialized hospital, Amhara regional state, Ethiopia, 2020.

S. No	Attitude Related questions	Frequency (%)		
		Agree	Not sure	Disagree
		n (%)	n (%)	n (%)
1	COVID-19 will finally be successfully controlled	199(50)	154(38.7)	45(11.3)
2	Strict measures can help win the battle against the COVID-19 virus	135(33.9)	203(51)	60(15.1)
3	Infection with the virus is associated with stigma	294(73.9)	45(11.3)	59(14.8)
4	Do you think that this disease is dangerous	366(92)	26(6.5)	6(1.5)
5	Do you agree that self-protection necessary for the protection of others	353(88.7)	35(8.8)	100(2.5)
6	Not everyone with COVID-19 will die	220(55.3)	80(20.1)	98(24.6)
7	Compliance with the Ministry of Health precautions will prevent the spread of COVID-19	298(74.9)	91(22.9)	9(2.3)
8	Washing hands is essential to protect me from COVID-19.	346(86.9)	50(12.6)	2(.5)
9	It is important to keep my distance from others, to avoid spreading COVID-19.	343(86.2)	47(11.8)	8.2)

As indicated in Table 4 above, from all factors, in a bivariate analysis, only five had a significant effect on poor attitude towards COVID-19 and from these factors, only two of them were significantly associated with poor attitude through multivariate analysis. The multivariate analysis indicated that those participants who can’t read and write, and who can read and write were 6.71 times (AOR = 6.71, 95% CI (2.78–16.16)) and 3.45 times (AOR = 3.45, 95% CI (1.56–7.81)) more likely to have poor attitude than those with educational status of “college and above” respectively. The odds of having a poor attitude in rural residents were 2 times (AOR = 2.03, 95% CI (1.14–3.61)) **(Table 5).**

**Table 5:** Factors associated with a poor attitude among outpatient service visitors at Debre Markos comprehensive specialized hospital, Amhara regional state, Ethiopia, 2020.

Variables	Poor attitude		COR (95%CI)	AOR (95%CI)	P-value
	Yes	NO			
Educational status					
Unable to read and write	46	27	11.35(5.25–24.54)	**6.71(2.78–16.16)**	0.01*
Read and write	35	47	4.96(2.35–10.49)	**3.45(1.56–7.81)**	0.01*
Elementary (1–8)	23	71	2.16(1.00–4.65	1.91(0.87(0.41)	0.1
Secondary (9–12)	6	51	0.78(0.27–2.22)	0.78(0.28–2.21)	0.64
College and above	12	80	1	1	
Residence					
Urban	54	220	1	1	
Rural	68	56	4.94(3.11–7.85)	**2.03(1.14–3.61)**	0.02*
Monthly income					
≤1000 ETB	49	54	3.53(2.08–5.98)	1.36(0.72–2.55)	0.34
1001–2500 ETB	35	74	1.84(1.07–3.15)	0.99(0.54–1.83)	0.98
≥2600 ETB	38	148	1	1	
Having television					
Yes	41	185	1		
No	81	91	4.02(2.56–6.31)	1.36(0.69–2.66)	0.36
Poor knowledge					
Yes	47	61	2.21(1.39–3.51)	0.85(0.47–1.52)	0.58
No	75	215	1	1	

Note: *Statistically significant.

### The practice of outpatient service visitors towards COVID-19

**Fig 4:**
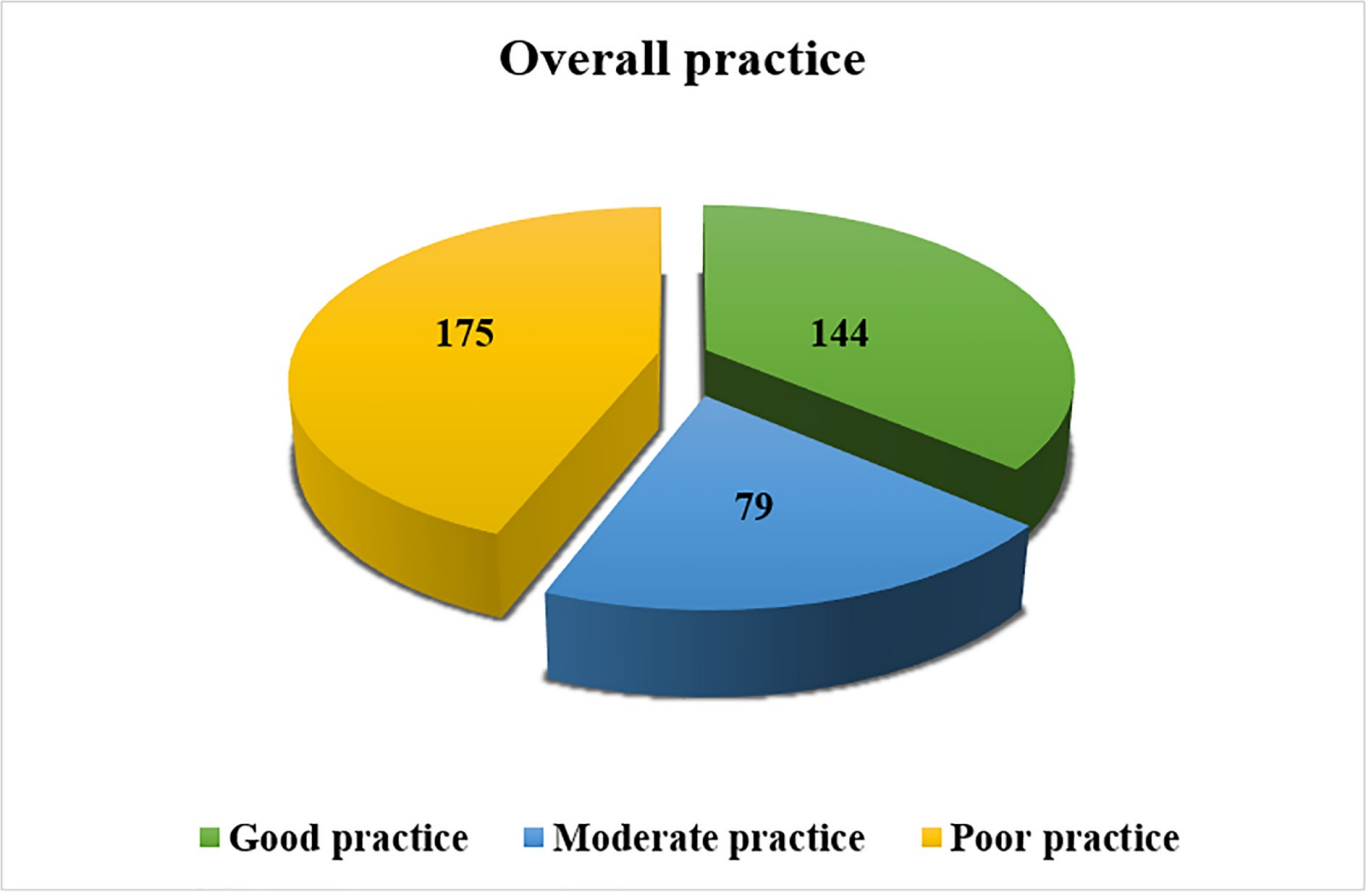
Level of overall preventive practice towards COVID-19 among outpatient service visitors in Debre Markos compressive specialized hospital, Amhara regional state, Ethiopia, 2020.

The prevalence of poor practice among outpatient service visitors was 175 (44%) and 144 (36.2%) of them had a good practice **(Fig 4).**

Among the participants, only 1/5^th^ (21.9%) of them reported that they were avoiding crowded places in the recent one week. Nearly half, 195 (49%) of them wearing a face mask during leaving their home. Two hundred twenty-three, (56%) practiced proper hand hygiene by frequently washing their hands or using hand sanitizer. Most, 322 (80.9%) of the participants had avoided handshaking **(Table 6).**

**Table 6:** Practice of preventive measure towards COVID-19 among outpatient service visitors at Debre Markos comprehensive specialized hospital, Amhara regional state, Ethiopia, 2020.

S. No	Preventive practice-related questions	Frequency (%)	
		Yes	No
		n (%)	n (%)
1	In recent one week have you gone to any crowded places?	311(78.1)	87(21.9)
2	In recent one week have you worn a mask when leaving home?	195(49)	2003(51)
3	In recent one week have you practiced proper hand hygiene by frequently washing your hands and using hand sanitizer?	223(56)	175(44)
4	Practice avoid touching eye, nose, mouth before washing hands.	228(57.3)	170(42.7)
5	Avoided proximity including while greeting (within 2 meters).	82(20.6)	316(79.4)
6	Stopped shaking hands while giving greeting.	322(80.9)	76(19.1)
7	Used cover/elbow for coughing/sneezing.	332(83.4)	66(16.6)
8	Listen and follow the direction of your state and local authorities.	277(69.6)	121(30.4)
9	Clean and disinfect frequently touched objects and surfaces.	156(39.2)	242(60.8)
10	Prefer to stay at home with an open window	182(45.7)	216(54.3)

The variables; residence, have not television and poor knowledge had a significant association with the poor practice, at p <0.05. Those who have not television were 2.64 times [AOR = 2.65, 95% CI (1.32–5.29)] more likely to have poor practice. Participants with poor knowledge about COVID-19 were 22.73 times [AOR = 22.73, 95% CI (10.5–49.2)] more likely to have poor practice. Outpatient service visitors from the rural area had 2.1 times [AOR = 2.08, 95% CI (1.88.-4.87)] higher likelihood of poor practice **(Table 7).**

**Table 7:** Factors associated with the poor practice among outpatient service visitors at Debre Markos Comprehensive Specialized Hospital, Amhara regional state, Ethiopia, 2020.

Variables	Poor practice		COR (95%CI)	AOR (95%CI)	P-value
	Yes	No			
Educational status					
Unable to read and write	45	28	3.87(2.02–7.42)	1.52(0.18–1.43)	0.20
Read and write	45	37	2.93(1.57–5.47)	1.44(0.17–1.21)	0.08
Elementary (1–8)	44	50	2.12(1.16–3.88)	1.63(0.79–3.33)	0.18
Secondary (9–12)	14	43	0.78(0.37–1.66)	1.08(0.47–2.45)	0.84
College and above	27	65	1	1	
Residence					
Urban	86	188	1	1	
Rural	89	35	5.56(3.49–8.87)	**2.08 (1.88.-4.87)**	0.04*
Monthly income					
≤1000 ETB	50	53	1.42(0.87–2.32)	0.62(0.23–1.32)	0.21
1001–2500 ETB	51	58	1.33(0.83–2.15)	1.15(0.60–2.18)	0.66
≥2600 ETB	74	112	1	1	
Having television					
Yes	63	163	1	1	
No	112	60	4.83(3.15–7.41)	**2.64 (1.32–5.29)**	0.01*
Poor knowledge					
Yes	97	11	23.96(12.19–47)	**22.73(10.5–49.2)**	0.01*
No	78	212	1		
Poor attitude					
Yes	68	54	1.98(1.29–3.06)	1.28(0.71–2.32	0.41
No	107	168	1		

Note: *Statistically significant.

## Discussion

The prevalence of poor (knowledge, attitude and practice) among the outpatient service visitors were 27.1%, 30.7% and 44%, respectively. This might show there was a limited access of information about the virus. Educational status; ‘can read and write” or below, rural residence and have not television had a significant association with poor knowledge. While, educational status of “can’t read and write”, and rural residence were significantly associated with poor attitude. Similarly, poor knowledge, rural residenc and have not television had a significant association with the poor practice. This finding shows that too much attention is needed for the entire community to give health education and awareness creation about KAP towards COVID-19.

The prevalence of poor knowledge in this study was higher than studies conducted in Ethiopia, 17% [15], Tanzania 15.6%, [16], Uganda 16.1%, [17], Malaysia 19,5% [18] and China 10% [14]. This difference may be due to the socioeconomic status and the availability of infrastructures. In this study, 167(42%) of study participants’ main sources of information was TV and/or radio, which was similar with a study done in Ethiopia [13] while in Egypt, the main source of information was social media (66.6%) [19]. This difference might be due to the difference in the study populations’ socio-economic status, educational status, and availability of the internet.

In this study, compared with participants who had college and above educational level, those who “can read and write” or below were significantly associated with poor knowledge. This was inline with studies conducted in Ethiopia [13], Nepal [20], Bangladeshi [21],Tanzania [16], and Egypt [19]. A study in China also reported that education of bachelor’s degree or lower were significantly associated with low knowledge score COVID-19 [14].

The present study showed that rural residents were three times more likely to have poor knowledge compared to urban residents. This could be due to a lack of access to information in rural areas, where there is a limited access to television or information sources like social media that help them to update themselves about COVID-19. This was similar to a study in Bangladesh [21] and Ethiopia [13]. Furthermore, most Ethiopian rural residents are unable to read and write with lack of health related information and reduced ability to understand health-prevention actions to prevent COVID-19. However, the main ways to access information in rural areas of Ethiopia are through family, friends, religious place and health care workers, which are not as timely as the means of acquiring information in urban areas. In this study, 390 (90%) of the study participants knew the main clinical symptoms of COVID-19. Among the participants, 342 (85.9%) reported that, touching the mouth, nose, or eye with unwashed hand was the means of COVID-19 transmission; while 311(78.1%) of them said that, corona spreads via respiratory droplets of infected individuals which was in line with a study conducted in Ethiopia [15]. In this study, 294 (74%) of the participants believed that infection with the virus is associated with a stigma which was lower than study done in Ethiopia, 83.3% but much higher than in Egypt, 22.7% [15,19]. This might be due to the difference in fear of its mortality and high communicability.

In this study, the prevalence of poor practice was found to be 175 (44%) which was higher than studies done in Nepal, 10% [20], and Uganda, 14.7% [17]. This may be due to the difference in the sources of information, frequency of media exposure and knowledge. In our study, 21.9% of the participants were avoiding themselves from crowded places. This was lower than studies conducted in Ethiopia, 38.1% [13], Malaysia, 83.4% [18], Nepal 94.9% [20], China 96.4% [14] and Tanzania, 77% [16]. This discrepancy might be due to the socio-economic, cultural, and religious differences. In this study, 195 (49%) of the participants wore a mask when leaving home which was higher than study in Ethiopia, 36.6% [13]; but lower than in China, (98.0%) [14]. This low practice of wearing a mask in this study might be due to the inability to afford the mask and lack of knowledge. In the present study patients with poor knowledge were more likely to have a poor practice which was in line with study done in China [14].

### Limitations of the study

The study was conducted in a health institution. As a result, there is a possibility of bias as underprivileged populations may not have been able to participate in the study. In addition, the cross-sectional nature of the study did not allow us to show the cause-effect relationship. Moreover, some of the questions included in the survey appear to be using language that is not very accessible for the average person. Some of the language appears to be technical and not well tailored to different language skills that might be present in the sample. This might have contributed to the patients’ answer (especially with the lack of knowledge).

## Conclusion

The study revealed that a high number of the study participants had poor knowledge, attitude, and practice towards COVID-19. Factors like; below elementary educational level, have not television, and rural residency were significantly associated with poor knowledge and attitude. Besides, participants with poor knowledge about COVID-19 had also a poor practice towards COVID-19 infection prevention. Therefore, to prevent COVID-19 spread, strict preventive and control measures must be implemented by the local government through banning public gatherings and enforcing people to follow all preventive measures of COVID-19. In addition, it is recommended that other reaserchers and responsible stakeholders to design a kind of project towards the pandemic.

## Supporting information

S1 File (DOCX)

## Abbreviations

AOR: Adjusted odds ratio
COR: Crude odds ratio
COVID-19: Coronavirus disease-2019
ARDS: Acute respiratory distress syndrome
KAP: Knowledge, attitude, practice
SARS: Severe acute respiratory distress syndrome
SARS–CoV-2: Severe acute respiratory distress syndrome coronavirus-2
WHO: World Health Organization
