# Supplementary material for: Knowledge, attitude and preventive practice towards COVID-19 and associated factors among outpatient service visitors at Debre Markos compressive specialized hospital, north-west Ethiopia, 2020
Source: PLoS One. 2021 Jul 15;16(7):e0251708. doi: 10.1371/journal.pone.0251708 (PMC8282282; doi:10.1371/journal.pone.0251708)
Supplement: S1 File — (DOCX) [file pone.0251708.s001.docx]

Supplementary material

**Single population proportion formula was used**

$$n=\frac{Z_{\frac{1-\alpha}{2}}^{2}(p)(1-p)}{d^{2}}$$

Where n**=** the desired sample size

p= the proportion of poor knowledge or attitude or practice = 50%.

Z= the standard normal score set at 1.961 (95% confidence interval)

d= the margin of error to be tolerated (5%) Therefore: -

n_i_ = (Zα/2)^2^ p (1-p) = (1.96)^2^ X (0.5x0.5) = 384

(0.05)^2^

**Debre Markos University**

**School of medicine**

**Consent form**

We, the investigators; Bekele Taye Feleke, Mengistu Zelalem Wale and Mesenbet Terefe Yirsaw; from Debre Markos University, School of Medicine, undertaking research work entitled “Knowledge, attitude and preventive practice toward COVID-19 among outpatient service visitors in Debre Markos compressive specialize hospital.” The aim of the research is “to assess knowledge, attitude and preventive practice towards COVID-19 among outpatient service visitors at Debre Markos compressive specialize hospital”, which will bring change in knowledge, attitude, and practice. Participating in the study does not involve any harms. Dear participants, the information that you will give us is quite useful to achieve the objective of the study. We would like to assure you that there is no risk or discomfort associated with your participation. To ensure confidentiality, your name will not be mentioned in the questionnaire and the information that you provide will be secured private and confidential and used for research purposes only. You have the full right to refuse or to interrupt the interview at any time. The interview will take about 40 minutes.

Are you voluntarily participate? 1. Yes 2. No

If No, respect the decision and thank her/him. Don’t force or reinforce an individual to participate. If yes continue to fill questions.

Interviewer’s code -------------

Data collectors name--------------------------------------------------------------sign-------------

Date of interview --------------------------

Supervisor’s name -----------------------------------------------------signature ------------------------

If you have any question and want to know more information regarding this study, you will contact the investigators by the following address**:**

**Investigators**

**Name: Phone No E-mail address**

Bekele Taye Feleke +251918072405 [bekeletaye414@gmail.com](mailto:bekeletaye414@gmail.com)

Mengistu Zelalem Wale +251920772670 [mengistubiot@gmail.com](mailto:mengistubiot@gmail.com)

Mesenbet Terefe Yirsaw +251910806714 [tmesenbet@gmail.com](mailto:tmesenbet@gmail.com)

**Questionnaires**

**I. Socio-demographic and related questions**

| No | **Questions** | **Answer** |
| --- | --- | --- |
| **1** | Age in complete years | --------years |
| **2** | Sex | 1. 1. Male 2. 2. Female |
| **3** | Educational Status | 1. 1. Unable to read and write 2. 2. Able to read and write 3. 3. 1-8^th^ grade 4. 4. 9-12th grade 5. 5. college and above |
| **4** | Religion | 1. 1. Orthodox 2. 2. Muslim 3. 3. Protestant 4. 4. Other |
| **5** | Occupation | 1. 1. Daily Laborer 2. 2. Merchant 3. 3. Private Employee 4. 4. Governmental Employee 5. 5. farmer 6. Others |
| **6** | Residence | 1. 1. Urban 2. 2. rural |
| **7** | Marital status | 1. 1. Single 2. 2. Married 3. 3.divorced 4. 4. widowed |
| **8** | Monthly income | -----------------Ethiopian Birr |
| **9** | Travel history | 1. 1. Yes 2. 2. No |
| **10** | Chronic medical illness | 1. 1. Yes 2. 2. No |
| **11** | Have you heard about COVID-19 | 1. 1. Yes 2. 2. No |
| **12** | If yes, Source of information about COVID | 1. 1. Social media 2. 2. Television/Radio 3. 3. Telecommunication 4. 4. Peer 5. 5. Religious place 6. 6. Health professional 7. others |
| **13** | Do you have a television in your home? | 1. 1. Yes 2. 2. No |
| **14** | Do you have a radio in your home? | 1. 1. Yes 2. 2. No |

**Part II: knowledge of COVID-19**

|  | Questions | Yes | No | I don’t know |
| --- | --- | --- | --- | --- |
| 1 | The main clinical symptoms of COVID-19 are fever, fatigue, dry cough, and body aches |  |  |  |
| 2 | Unlike the common cold, stuffy nose, runny nose, and sneezing are less common in persons infected with the COVID-19 virus. |  |  |  |
| 3 | Currently, there is no effective cure for COVID-19, but early symptomatic and supportive treatment can help most patients recover from the infection. |  |  |  |
| 4 | Not all persons with COVID-19 will develop into severe cases. Only those who are elderly and have chronic illnesses are more likely to be severe cases. |  |  |  |
| 5 | Eating or touching wild animals would result in the infection by the COVID-19 virus. |  |  |  |
| 6 | Persons with COVID-19 cannot infect the virus to others if they do not have a fever. |  |  |  |
| 7 | The COVID-19 virus spreads via respiratory droplets of infected individuals |  |  |  |
| 8 | One way of prevention of COVID 19 is not touching the eye, nose by unwashed hands |  |  |  |
| 9 | Ordinary residents can wear face masks to prevent the infection by the COVID-19 virus. |  |  |  |
| 10 | Children and young adults do not need to take measures to prevent the infection by the COVID-19 virus. |  |  |  |
| 11 | To prevent infection by COVID-19, an individual should avoid going to crowded places. |  |  |  |
| 12 | Isolation and treatment of people who are infected with the COVID-19 virus are effective ways to reduce the spread of the virus |  |  |  |
| 13 | People who have contact with someone infected with the COVID-19 virus should be immediately isolated in a proper place. |  |  |  |

**Part III: Attitudes towards COVID-19**

|  | Questions | Response | | |
| --- | --- | --- | --- | --- |
| 1 | Do you agree that COVID-19 will finally be successfully controlled? | Agree | Not sure | Disagree |
| 2 | Do you have confidence strict measures can help win the battle against the COVID-19 virus? | Agree | Not sure | Disagree |
| 3 | Infection with the virus is associated with stigma (for example; the infected persons feel ashamed because people are afraid of and avoid them) | Agree | Not sure | Disagree |
| 4 | Do you think that this disease is dangerous | Agree | Not sure | Disagree |
| 5 | Do you agree that self-protection necessary for the protection of others | Agree | Not sure | Disagree |
| 6 | Not everyone with COVID-19 will die | Agree | Not sure | Disagree |
| 7 | Compliance with the Ministry of Health precautions will prevent the spread of COVID-19 | Agree | Not sure | Disagree |
| 8 | Washing hands is essential to protect me from COVID-19. | Agree | Not sure | Disagree |
| 9 | It is important to keep my distance from others, to avoid spreading COVID-19. | Agree | Not sure | Disagree |

**Part III: practice towards COVID-19 prevention.**

| S.no | Questions | Response | | |
| --- | --- | --- | --- | --- |
| 1 | In recent one week have you gone to any crowded places (meetings, religious activities, market)? | Yes | No |  |
| 2 | In recent one week have you worn a mask when leaving home? | Yes | No |  |
| 3 | In recent one week have you practiced proper hand hygiene by frequently washing their hands and using hand sanitizer | Yes | no |  |
| 4 | Have you practiced avoid touching eye, nose, mouth before washing hands | Yes | no |  |
| 5 | Have you avoided proximity including while greeting (within 2 meters) | Yes | no |  |
| 6 | Have you stopped shaking hands while giving greeting | Yes | no |  |
| 7 | Have you used cover /elbow for coughing/sneezing | Yes | no |  |
| 8 | Do you listen and follow the direction of your state and local authorities? | yes | No |  |
| 9 | Do you clean and disinfect your hands frequently after touching objects and surfaces | yes | No |  |
| 10 | Do you prefer to stay at home, in a room with the window open currently? | yes | no |  |
